# GENOME-WIDE META-ANALYSIS SUPPORTS HETEROGENEITY IN POLYGENIC ARCHITECTURE OF DISABILITY

**DOI:** 10.1093/geroni/igz038.335

**Published:** 2019-11-08

**Authors:** Alexander Kulminski, Chansuk Kang, Yury Loika, Eric Stallard, Irina Culminskaya

**Affiliations:** Duke University, Durham, North Carolina, United States

## Abstract

Disability and frailty increase steeply with age after midlife. They comprise broad, non-specific aging-related health/well-being declines. Understanding the molecular mechanisms of these declines could help in combating processes related to intrinsic aging. We performed genome-wide meta-analysis of disability using data on N=12,550 disabled individuals of Caucasian ancestry from five independent studies with N=24,068 genotyped participants. Disability was measured using the Activities of Daily Living (ADL) scale. A subject was considered disabled if he/she had at least one ADL impairment. All subjects in our study were aged 50+ years. The analysis followed a discovery-replication strategy using two studies as discovery samples and three others as replication samples. At the discovery stage, we selected SNPs at nominal significance (p<0.05) and evaluated their associations with disability in the replication samples. Meta-analysis across all studies identified 30 SNPs (24 loci) at p<10e-4. SNPs from four loci (chromosomes 2, 8, and 12) attained suggestive significances at p<10-5. We constructed two polygenic risk scores (PGRS) using these 30 SNPs whose minor alleles were positively (19 SNPs) and negatively (11 SNPs) associated with disability, PGRS_p and PGRS_n, respectively. Meta-analysis across all studies identified strong effects for PGRS_p (beta=0.436, p=1.18×10e-32) and PGRS_n (beta=-0.426, p=8.74×10e-19). The associations for the PGRSs observed in two discovery studies were replicated in three independent studies. The lack of genome-wide significant effects of individual SNPs combined with the highly significant effects of the PGRSs shows that, in line with the heterogeneous origin of disability, its genetic architecture is of highly heterogeneous polygenic origin.

